# Psychological Responses of Hungarian Students during the First Wave of the COVID-19 Pandemic

**DOI:** 10.3390/ijerph191811344

**Published:** 2022-09-09

**Authors:** Kata Morvay-Sey, Melinda Trpkovici, Pongrác Ács, Dávid Paár, Ágnes Pálvölgyi

**Affiliations:** 1 Institute of Physiotherapy and Sport Sciences, Faculty of Health Sciences, University of Pécs, Vörösmarty u.3., 7621 Pécs, Hungary; 2 Doctoral School of Health Sciences, Faculty of Health Sciences, University of Pécs, Vörösmarty u.4, 7621 Pécs, Hungary

**Keywords:** COVID-19 pandemic, perceived stress, mindfulness, well being

## Abstract

(1) Background: Changes in daily life and academic training has led to uncertainty in the higher education student population during COVID-19. The goal of the study was to examine the impacts of the pandemic on Hungarian students. (2) Methods: A cross-sectional study was conducted by using self-report questionnaires collected in Google Forms. Eight-hundred-and-twenty-seven students (25.29 ± 8.09) took part anonymously. The respondents rate their overall physical and mental health on a 5-point Likert scale and validated scales were used: Well Being Index (WHO-5); Mindfulness Attention and Awareness Scale (MAAS); and Perceived Stress Scale (PSS-14). Statistical analyses were performed with IBM SPSS 24.0, results were considered at a significance level *p* ≤ 0.05. (3) Results: Positive correlation was found between MAAS and WHO-5 (r = 0.363, *p* < 0.001) negative correlation between MAAS and PSS-14 (r = −0.448, *p* < 0.001), and negative correlation between WHO-5 and PSS-14 (r = −0.671, *p* < 0.001). Females had higher PSS-14 mean score (32.51 ± 10.16) than males (27.71 ± 10.19; *p* < 0.001; Z = −5703), males (60.92 ± 12.10) had higher MAAS level than females (57.31 ± 12.51; *p* < 0.001; Z = −3589). No difference was found in gender regarding WHO-5 mean scores. Athletes (7.03 ± 3.27) differ significantly from non-athletes (6.00 ± 3.04) in WHO-5 (*p* < 0.001; Z = −4.349) and MAAS level (*p* = 0.012; Z = −2.498), but showed no difference in PSS-14 (*p* = 0.101; Z = −1.641). Students rated mental (3.01 ± 0.99) worse than physical health (3.49 ± 0.98; *p* < 0.001, r = 0.426) and the narrowing of social relationships worse (3.83 ± 1.26) than physical (*p* < 0.001, r = −0.212) and mental health (*p* < 0.001, r = −0.408). Females had worse mental health (2.96 ± 9.94) than males (3.20 ± 0.99; *p* = 0.003; Z = −2.924) and rated the narrowing of social relationships worse (3.90 ± 1.23) than males (3.59 ± 1.35; *p* = 0.006; Z = −2.730). (4) Conclusions: The pandemic has negatively impacted students, and it may have long-term consequences on their mental and physical health and education.

## 1. Introduction

COVID-19 is the third major coronavirus outbreak over the past 20 years that has had a substantial socioeconomic impact, but the first in the 21st century to affect countries across all continents except Antarctica [1]. The pandemic has rapidly become the most significant public health crisis of the 21st century and the most important factor in our life that is having a significant impact on all aspects of society, including mental and physical health. The United Nations Secretary-General has called the COVID-19 pandemic the largest humanitarian crisis since the Second World War, and authors pointed out that since the Second World War, the SARS-CoV-2 coronavirus epidemic has been the longest-lasting crisis affecting all of humanity globally [2].

The fear of becoming infected with the virus has resulted in significant changes in our daily lives. Previous lifestyle, change of daily routine and working conditions (change of work schedule and leisure habits, unemployment), a decrease in activity (physical, social, tourism, and traveling), lack of free movement or limitation of movement (access restrictions, stay at home orders, quarantine, changes in sports activity), the possibility of working from home (home office, hybrid work), compulsory full or partial digital home education for school children and university students, the lack of contact-based social life and personal contact within family members and friends—this all had a significant impact on the biopsychosocial status, physical and mental health, and well-being of individuals. Direct and indirect psychological effects have had an impact on the whole society. Based on the relevant literature, it is visible that certain more vulnerable risk groups are of greater interest to the researchers. Such groups are the elderly, juveniles (students) and higher-education students, employees, individuals with previous mental illness, health professionals, and infected individuals.

The rigid curfew during the first wave of the pandemic lasted in Hungary from 28 March to 4 May 2020. One was allowed to go outside only if it was necessary (go to work, do the shopping). Except for food stores, drug stores, pharmacies, and petrol stations, all other shops and educational institutions remained closed. The Hungarian Government ordered a ban on visiting the university and academic institutions from 12 March 2020 to prevent the spread of the virus. A 2-day break period without education (12–13 March) was ordered first, then the spring holiday was brought forward (16–20 March) to have enough time to prepare for online education. Only distance learning (online, e-learning) was allowed at universities from the 23 March until the end of the semester [3].

Academic youth is an interesting group of people for researchers, as they have never experienced social disasters before. The novelty and unknown nature of strict quarantine restrictions or curfew, which affected personal freedom (curfew, quarantine, social isolation, opening hours of shops, gyms, theatres, restaurants, lack of events, contact restrictions in nursing homes, residential homes, etc.) and the growing economic crisis (existential problems and unemployment) caused anxiety and mental instability. In the case of university students, these rapid and unknown changes in daily life and the radical changes in the nature of the educational experience have led to uncertainty.

Observations of university students across the world pointed out that students experienced increased stress level, anxiety, fear, and depressive symptoms as a result of the uncertainty of university education, academic success, technological concerns of online courses, social isolation, decreased family income, and future careers and employment prospects [4,5,6,7]. For instance, Gogoi et al., (2022) aimed to understand how pandemic experiences have affected well-being by conducting in-depth interviews with 34 undergraduate students. The authors identified major factors that have influenced students’ mental health and well-being: isolation, bereavement, academic concerns, financial worries, and support, coping, and resilience [8]. They realized the importance of support from family, friends, and the institution for maintaining the mental health and well-being of students during this extremely stressful time. Gómez-García et al., (2022) investigated 1873 university students in Spain (Andalusian University) and found results that reflected the strong negative impact that the pandemic had, especially on the level of life satisfaction and the indices of depression, anxiety, and stress of students [9]. Wang et al. (2020) carried out an online survey, which was conducted among undergraduate and graduate students recruited from Texas A&M University via email. The survey consisted the Patient Health Questionnaire-9 and the General Anxiety Disorder-7—for depression and anxiety, and additional multiple-choice and open-ended questions regarding stressors and coping mechanisms specific to COVID-19. Among the 2031 participants, 48.14% (*n* = 960) showed a moderate-to-severe level of depression, 38.48% (*n* = 775) showed a moderate-to-severe level of anxiety, and 18.04% (*n* = 366) had suicidal thoughts. A majority of participants (*n* = 1443, 71.26%) indicated that their stress/anxiety levels had increased during the pandemic. Less than half of the participants (*n* = 882, 43.25%) indicated that they were able to cope adequately with the stress related to the current situation [10]. Fu et al., (2021) found that about two-fifths of Chinese college students experienced anxiety symptoms during the COVID-19 epidemic. The authors pointed out that timely and appropriate psychological interventions for college students should be implemented to reduce the psychological harm caused by the COVID-19 epidemic [11].

The goal of the current study is to examine the difference in psychological and physical health on Hungarian higher education students during the first wave of the COVID-19 pandemic in the spring semester of the academic year 2019/2020. We aimed to explore certain psychological effects of the coronavirus pandemic (level of trait mindfulness, perceived stress and well-being, subjective mental and physical status, the narrowing of social relationships) on students and identify potential sociodemographic protective/risk factors. Several research conducted among university students found evidence of an increasing level of anxiety, depression, distress, and mental health disorders during the pandemic, but only a few studies have been published so far where the authors examined mindfulness and its correlation to other intrapsychic characteristics in this stressful period [12,13]. Mindfulness means paying attention to the present moment (conscious awareness) and doing it non-judgementally. Individuals who have higher mindfulness levels report better perceived health, trait mindfulness helps people to handle distress and to improve their life satisfaction, and dispositional mindfulness promotes well-being [14]. The higher the mindfulness level is, the better the subjective well-being of the individual is. Mindfulness may promote effective coping during periods of uncertainty crisis [15,16]. Findings of a research indicated that mindfulness enhances well-being and helps in coping with stressful situations such as the COVID-19 pandemic [17].

Because of the quick and radical changes in the learning and teaching process, and because of the rapid shift to online courses, we decided to investigate the students’ opinion about the online learning situation.

## 2. Materials and Methods

### 2.1. Study Population

A cross-sectional, observational cohort study was conducted with consecutive sampling using validated self-report questionnaires via Google Forms.

Data collection was carried out at the University of Pécs (UP), Hungary. All the respondents were adults and active students (BSc, MSc, Ph.D., and postgraduate) at 1 of the 10 faculties of the University of Pécs in the spring semester of the academic year 2019/2020. The data were obtained through a web-based comprehensive questionnaire method (with a free Google account-Google Forms) to avoid possible infections. Eight-hundred-and-twenty-seven respondents filled out the self-report questionnaire from 5 to 31 May 2020 during the first wave of the COVID-19 pandemic when continuing their studies online (from 12 March 2020). The mean age of the students was 25.29 ± 8.09 (min. age: 18 max. age: 63 years, median: 22 years). Of the sample, 21.64% (*n* = 179) were male and 78.36% (*n* = 648) were female. Of the respondents, 46.67% live in a city, 28.78% in a town, 17.78% in a village, and only 6.77% in the capital. The sample included 10.88% married, 31.44% cohabitated, and 57.68% single participants. Overall, 88.27% had no child, 5.32% had two children, 3.99% had one child, and 2.42% of the respondents had three children.

### 2.2. Measures

Socio-demographic questions and sporting habits: The questionnaire was originally divided into nine sections. Socio-demographic characteristics of the sample were obtained through general questions regarding gender, place of living, marital status, number of children, faculty, and main activity. We asked the respondents about their sporting habits (doing sport regularly or not), the frequency and duration of sports activity, and exercising before and during quarantine. To measure the internal consistency of the used validated scales, we tested the Cronbach alfa coefficient which, in the case of MAAS, was α = 0.862; PSS-14 α = 0.910; and WHO-5 α = 0.815. The internal reliability of the questionnaire (total), as well as the items, are also adequate.

Self-reported subjective physical and mental health with regards to social isolation: We measured the subjective mental and physical health with self-assessment questions: “How do you rate your overall mental health/physical health during the COVID-19 curfew?”. The respondents had to rate their overall physical and mental health on a 5-point Likert scale, where a score of 1 meant “the worst (very bad)” and a score of 5 meant “the best (very good)”. To measure the judgment of social isolation, students were asked to describe how they rate the narrowing of their social relationships during the curfew on a 5-point Likert scale: “How much were you bothered by the narrowing of your social relationships (friends, acquaintances, colleagues) at the time of the COVID-19 curfew? (1 = not at all; 5 = heavily).

Questions about academic life and the pandemic’s effect on their experiences regarding distance learning: Students were asked about their academic life and the pandemic’s effect on their experiences regarding teaching and learning online in four questions. The mental burden of online learning’s technical background (synchronous, asynchronous), the difficulty of mastering theoretical and practical subjects, and the depersonalization of studies with technical devices of communications were measured by close-ended questions.

Well-Being Index (WHO-5): 

The 5-item World Health Organisation (WHO-5) Well Being Index [18] WHO-5 [19] was derived from the WHO-10 [20], developed as a generic scale without any diagnostic specificity. It was validated in Hungarian by Susánszky et al. [21].

This instrument measures subjective psychological well-being of the past 2 weeks with five items, and it is a good screening tool for depression. The Hungarian version, dissimilar to the original scale, uses a 4-point Likert scale. It has a single factor structure; its total score must be calculated by adding the single items’ points. The higher scores mean better well-being. The WHO-5 has been shown to have high reliability and validity across many different samples in different countries [18].

The Perceived Stress Scale (PSS-14) developed by Cohen et al. [22] is one of the most commonly used scale to measure stress level. The reliability and validity of the original questionnaire were verified, and the scale is widely used to examine the effects of chronic stress in various patient and healthy populations [23]. The questionnaire shows a moderately strong correlation with the WHO-5 well-being scale. We used the Hungarian validated version of the PSS-14, which was developed by Stauder and Konkolÿ Thege [24]. 

Mindful Attention Awareness Scale (MAAS) was developed by Brown and Ryan [25], and it was validated in Hungarian (MAAS-H) by Simor et al. [26]. The scale measures dispositional mindfulness, consists of 15 items, forming a single factor structure. Each of the items is stated inversely using a 6-point Likert scale. The scale’s total score can be calculated by adding the single items’ points. Higher scores indicate higher levels of trait (dispositional) mindfulness.

### 2.3. Statistical Analyses

Data preparation, aggregation, cleaning, and all statistical analysis were performed with Microsoft Excel 2010 and the Statistics Package for Social Sciences (SPSS) 24.0. programs; the results were considered significant if *p* ≤ 0.05. The Kolmogorov–Smirnov test was used to examine the normality of the data, and in case of a non-normal distribution, we used the non-parametric Mann–Whitney U-test, and Kruskal–Wallis test, depending on the number of sample groups. Pearson’s correlation was used to correlate aggregate scores and Spearman’s correlation for Likert scales. 

### 2.4. Ethical Approval

Ethical approval was granted for the study by the Hungarian Scientific and Research Ethics Committee (TUKEB IV/4599-2/2020/EKU) and Regional Research Ethics Committee (REKEB) Participants were informed about the research aim and methods before signing the informed consent form. The investigation conforms to the principles outlined in the Declaration of Helsinki.

## 3. Results

### 3.1. Correlation between the Mean of the Psychological Scale Total Scores (MAAS, WHO-5, PSS-14)

To examine the psychological factors, we used Pearson’s correlation and found a significant positive correlation between the dispositional mindfulness (MAAS) total score and well-being (WHO-5) total score (r = 0.363, *p* < 0.001), a significant negative correlation between dispositional mindfulness (MAAS) total score and perceived stress (PSS-14) total score (r = −0.448, *p* < 0.001), and significant negative correlation between well-being total (WHO-5) score and perceived stress (PSS-14) total score (r = −0.671, *p* < 0.001). The perceived stress total score’s mean of the university student sample was 31.47 ± 10.35, the dispositional mindfulness (MAAS) total score’s mean was 58.09 ± 12.50, and the WHO-5 well-being total score’s mean was 6.72 ± 3.24 during the first wave of the COVID-19 pandemic (Table 1).

### 3.2. Differences between Perceived Stress (PSS-14), Trait Mindfulness (MAAS), and Well-Being (WHO-5) Total Scores Regarding Gender, Sporting Attitude, and Marital Status

#### 3.2.1. Gender

**Figure 1:**
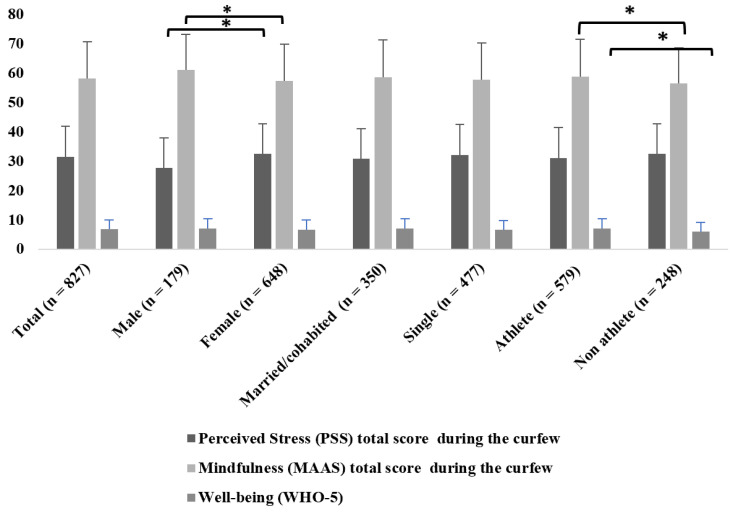
Differences of perceived stress (PSS-14), trait mindfulness (MAAS), and well-being (WHO-5) total scores regarding gender, sporting attitude, and marital status (* *p* ≤ 0.001 mean ± standard deviation).

Concerning stress levels, females had a significantly higher mean of perceived stress total score (32.51 ± 10.16) than males (27.71 ± 10.19; *p* < 0.001; Z = −5.703) during the first wave of the COVID-19 pandemic, and males had a significantly higher mean of mindfulness total score (60.92 ± 12.10) than females (57.31 ± 12.51; *p* < 0.001; Z = −3.589). There was no significant difference between the genders in well-being total score (*p* = 0.339; Z = −0.957); the mean total score of males was 6.92 ±3.34 and the mean score of females was 6.67 ± 3.34 (Figure 1).

#### 3.2.2. Marital Status

To measure the assumed protective factor of social relationships, we divided the sample into two groups regarding marital status. People who are married or cohabitated (*n* = 350; 30.78 ± 10.30) did not differ statistically from the group of singles (*n* = 477; 31.98 ± 10.37) in the total mean score of perceived stress (*p* = 0.114; Z = −1.579). There is no significant difference between the groups regarding mindfulness total mean score (married/cohabitated 58.53 ± 12.64; single 57.77 ± 12.41; *p* = 0.378; Z = −0.881), and in the WHO-5 total mean score (*p* = 0.217; Z = −1.235) between singles (6.56 ± 3.09) and married/cohabitated individuals (6.94 ± 3.42) (Figure 1).

#### 3.2.3. Sporting Attitude

We divided the sample into two groups based on sporting attitudes to measure its effect on mental condition and well-being. Athletes were defined as people exercising regularly (three times/week) and non-athletes as not doing spots regularly.

We found a significant difference between athletes (7.03 ± 3.27) and non-athletes (6.00 ± 3.04) in the total mean score of well-being (*p* < 0.001; Z = −4.349) and trait mindfulness total mean score (*p* = 0.012, Z = −2.498), but no significant difference was found regarding perceived stress level total mean score (*p* = 0.101; Z = −1.641). The trait mindfulness total mean score was significantly higher for athletes (58.77 ± 12.68) than for non-athletes (56.51 ± 11.96). The perceived stress level showed no significant difference in the total mean score (athletes: 31.06 ± 10.35; non-athletes 32.43 ± 10.32) (Figure 1).

### 3.3. Differences of Subjective Mental and Physical Health Status, Assessment of Social Relations Regarding Gender, Sporting Attitude, and Marital Status

The students did not consider their mental and physical health status to be very bad during the first wave of the pandemic. The mean score of the self-rated mental health (1–5 Likert scale) was 3.01 ± 0.99 in the sample, and the mean score of the physical health value was 3.49 ± 0.98. The narrowing of social relations was evaluated by a mean value of 3.83 ± 1.26.

Of the students, 5.6% (*n* = 46) reported that their mental health was the worst (very bad)” during the curfew but 5.9% (*n* = 49) chose the other end of the Likert scale, so they consider their mental status as being “the best (very good).” Only 2.3% (*n* = 19) of the students answered that their physical health status was “the worst” during the curfew, however, 15% (*n* = 124) of the respondents felt their physical health as “the best” possible. Of the students, 42% (*n* = 347) rated the limitation of social relations with the highest value (5), and 5.9% (*n* = 49) felt not to be restricted regarding their social relationships. It is visible that a significant proportion of the students perceived the narrowing of the relationships as a determining factor in their lives. Using Spearman’s correlation, we found a significant difference between the means of self-rated mental and physical health status and social relationships. The students rated their mental health status (3.01 ± 0.99) significantly worse than their physical health status (3.49 ± 0.98) (*p* < 0.001, r = 0.426). However, they felt the narrowing of social relationships significantly worse (3.83 ± 1.26) than their physical health (*p* < 0.001, r = −0.212) and mental health (*p* < 0.001, r = −0.408).

#### 3.3.1. Gender

Concerning mental health status, females reported significantly worse mental health total score (2.96 ± 9.94) than males (3.20 ± 0.99; *p* = 0.003; Z = −2.924) during the first wave of the COVID-19 pandemic. Females rated the narrowing of social relationships significantly more intensively (3.90 ± 1.22) than males (3.59 ± 1.35; *p* = 0.006; Z = −2.730). There was no significant difference between genders regarding subjective physical health status (*p* = 0.929; Z = −0.089); the mean value of both genders was the same, 3.49 ( ± 0.944 males; ±0.996 female).

#### 3.3.2. Marital Status

Married or cohabitating students did not differ significantly from the group of singles in the examined variables. There is no difference between the groups regarding mental health (married/cohabitated 3.07 ± 1.00; singles 2.97 ± 0.99; *p* = 0.268; Z = −1.108), physical health (married/cohabitated 3.47 ± 0.99; singles 3.5 ± 0.976; *p* = 0.601; Z = −0.524) and judging the narrowing of social relationships (married/cohabitated 3.78 ± 1.23; singles 3.87 ± 1.28; *p* = 0.193; Z = −1.303).

#### 3.3.3. Sporting Attitude

We found a significant difference between athletes (3.61 ± 0.96) and non-athletes (3.20 ± 0.96) regarding subjective physical health status (*p* < 0.001; Z = −5.623). Athletes have rated their health better. A significant difference was found between the two groups regarding the narrowing of social relationships (*p* = 0.001, Z = −3.39): athletes reported higher scores (3.92 ± 1.24) than non-athletes (3.62 ± 1.29). No significant differences were found regarding mental health status (athletes: 3.03 ± 1.01; non-athletes 2.98 ± 0.96; *p* = 0.482; Z = −0.703).

### 3.4. Results Related to University Studies, Academic Work

Because of this special sample, we were curious about how the university students felt and experienced the sudden change in their studies. We asked them about the mental burdens of the e-Learning (distance learning) platforms’ technical background, the difficulty of mastering theoretical/practical subjects, and the depersonalization of their studies by technical communication devices. Of the students, 30.89% (*n* = 255) answered “yes” to the question “Do the technical challenges of attending university courses make you nervous/ stressed?”, 69.17% (*n* = 572) said “no”, so the majority did not find the technical challenges distracting.

However, those who felt the technical challenges of the eLearning (distance learning) stressful had significantly worse results in all measured variables than those who did not find it stressful. Measured values and significance are presented in Table 1. The group that answered to be stressed about the technical challenges, felt their subjective physical and mental health significantly worse, detected the narrowing of social relationships significantly more, and had significantly higher stress level and worse well-being level than the other group. Those who answered not to be stressed about the technical requirement of learning had a significantly higher mean score of trait mindfulness. 

The students were asked the following: “Do you find it difficult/stressful to communicate with the professors and fellow students during online classes through technical devices?”. Of students, 40.26% (*n* = 333) thought that it was difficult to communicate online with professors and other students, however, the majority (59.74%, *n* = 494) felt the opposite. Respondents who did not find this form of communication stressful rated their subjective physical and mental health significantly better, detected the narrowing of social relationships significantly less, and had significantly lower stress level and better well-being level. People not stressed about online communication had significantly higher mindfulness levels (Table 1).

Online education was unusual in many practice-oriented training courses (e.g., sport science training, health professional training, etc.). To measure the students’ opinion, we asked the following question: “What do you think the learning process is like for practical subjects (seminars, practical lessons) in form of distance learning?” Six response options were available, and the respondents could choose only one of them. A majority, 35.60% (*n* = 294) of the students answered that it was harder and much more difficult to acquire practical knowledge because it was shared theoretically what you should do in practice. Overall, 20.30% (*n* = 168) answered learning the practice only in theory is much more difficult because it requires independent practice and training at home; 17.00% (*n* = 141) answered that practicing at home is an unfulfillable task for them (because of a lack of staff and equipment); 6.30% (*n* = 52) felt to absolve the practice-based courses this way more easily; 12.10% (*n* = 100) did not feel any changes compared to previous experience (personal attendance); and 8.70% (*n* = 72) could not decide on the question. We found significant differences regarding perceived stress (χ^2^ = 78.171; *p* < 0.001) mindfulness (χ^2^ = 11.781; *p* < 0.001), well-being (χ^2^ = 45.789; *p* < 0.001), subjective rated mental health (χ^2^ = 34.690; *p* < 0.001), and the narrowing of social relationships (χ^2^ = 36.460; *p* < 0.001), but we did not find significant difference regarding subjective physical health between the groups based on the given answers (6 groups) (χ^2^ = 9.579; *p* = 0.088). Students who felt easier to absolve the practice-based courses online, those who did not feel any changes compared to a previous period, and those who could not decide the question, had better values on the scales (higher MAAS score, WHO-5, higher subjective mental and physical health mean score, but lower PSS-14 score and lower mean score on the subjective rating of the narrowing of the social relations) than those who felt much more difficult to acquire practical knowledge, who answered learning the practice only in theory is much more difficult, and who answered practicing at home is an unfulfillable task (Table 2).

Regarding lectures, the students could choose only one of four response options for the following statement: “In the case of theoretical lectures, do you consider that learning the course material was successful during distance learning?”. In response, 42.70% (*n* = 353) answered “to learn the theoretical subjects is more difficult because it takes more time to process the material”, 29.60% (*n* = 245) chose that learning online is “easier because online recorded lectures can be replayed”, 19.30% (*n* = 160) did not feel any changes compared to previous experiences, and 8.30% (*n* = 69) could not decide on the question. Measuring the difference between the groups based on the given answers (four groups), we found significant differences with the Kruskal–Wallis test regarding all measured scales (perceived stress (χ^2^ = 83.981; *p* < 0.001), mindfulness (χ^2^ = 15.263; *p* < 0.002, well-being (χ^2^ = 72.975; *p* < 0.001), subjectively rated physical (χ^2^ = 11.069; *p* < 0.001) and mental health (χ^2^ = 84.281; *p* < 0.001), and narrowing of social relationships (χ^2^ = 34.161; *p* < 0.001).

Students who felt that mastering the theoretical subjects is more difficult, had lower MAAS score, WHO-5 score, PSS-14 score, and subjective mental and physical health mean score, but higher mean score on a subjective rating of the narrowing of the social relations than those who could absolve the lectures easier, those who did not feel any changes, and those who could not decide on the question (Table 2).

## 4. Discussion

In our cross-sectional study, we compared a higher education student sample divided into groups through different aspects. We examined the level of mindfulness, perceived stress, well-being, subjective physical and mental health, and opinion about the narrowing of social relationships. As one of the most influential and important factors in daily life of the studied group, we examined the perceptions of changes related to university studies/higher education training. The results showed that students experienced negative impacts of COVID-19 in the first wave of the epidemic. In our study, we examined the difference in perceived stress, trait mindfulness, and well-being total scores regarding gender, marital status, and sporting attitude, and we described results related to university studies and academic work. In this chapter, we follow the logic of this division and give conclusions from our results along the mentioned categories.

First of all, we measured the correlation between the mean of the psychological scale total scores (PSS-14, MAAS, WHO-5). We detected a significant negative correlation between well-being and perceived stress. Stauder and Konkolÿ Thege [24] found a moderately strong negative correlation between well-being (WHO-5) and perceived stress (PSS-14), similarly to our research. Kapoor et al. (2021) found an inverse association between perceived stress and psychological well-being during COVID-19 [27]. We found a significant positive correlation between dispositional mindfulness and well-being, and a significant negative correlation between dispositional mindfulness and perceived stress levels. Sweeny et al. (2020) found in their research that mindfulness was associated with better well-being during stressful circumstances in China [16]. Prior studies [28,29] found that mindfulness significantly correlates with well-being. Regarding mindfulness, our results are similar to Mettler et al. [30], who reported the protective role of trait mindfulness against problematic behavior (e.g., pathological video gaming). They also used Mindfulness Attention and Awareness Scale and found that dispositional mindfulness and elements of subjective well-being are positively correlated with each other. The higher the mindfulness level is, the better the subjective well-being of the individual is. Arslan [31] found a significant and positive correlation between mindfulness and subjective well-being. Belen [32] reported that mindfulness mitigated the impacts of coronavirus, fear, anxiety, and depressive symptoms. Conversano et al. [17] revealed that mindfulness is a significant predictor of psychological distress in adults; high mindfulness improves well-being to deal with the COVID-19 pandemic.

Examining a Hungarian representative sample (*n* = 1200) during first wave restrictions in Hungary (May 2020), the mean value of perceived stress was 22.10 ± 7.16. A 9.37-point difference was found compared to our results, which means that students felt this period was more stressful than the average [33,34,35]. This period was at the end of the university semester and at the beginning of the exam period, which is a very busy period in academic teaching. Szabó, Pukánszky, and Kemény [36] used validated psychological questionnaires to measure epidemic-related mental effects, including perceived stress levels (PSS-10), on a Hungarian sample of 431 people during the first wave in May 2020. The scores of their respondents were significantly higher than certain studies’ measured stress scores. 

Regarding gender, no significant difference in well-being level was found in our research. White et al. [37] measured the impact of the COVID-19 pandemic on mental health and well-being in a convenience sample of 600 UK adults, using a cross-sectional design. They found no significant differences in the level of well-being between males and females. The average score (WBI-5) of the Hungarian population was 9.63 points in the previous Hungarostudy survey [38]. We found lower quality of life scores (6.72 ± 3.24) during curfew restrictions, which is also lower when examined by gender than the average score of the Hungarostudy 2013. A 7.67 points well-being index was measured on our previous Hungarian representative sample during the restrictions, so students reached 0.95 points lower mean scores in our sample. Female respondents over 50 had the lowest average score (7.06), while the best quality of life was reported by young adults between the ages 18–29 (8.32). Students who are young adults—so the same age population as in our sample—reached only 6.72 points [33]. 

Concerning perceived stress levels, females had significantly higher perceived stress total score than men in our sample. Szabó, Ábel, and Boros [39] measured the perceived stress level of 1.552 respondents in Hungary in the middle of April 2020. They found that women reported more stress than men. Kowal et al. [40] compared the stress levels (PSS-14–10) between 30 March and 6 April 2020 in 27 countries and areas and found that women were more stressed than men. Gamonal-Limcaoco et al. [41] observed significantly higher scores of stress (PSS-10) among women, and Qiu et al. [42] found that women reported greater psychosocial distress than men. Men had significantly higher dispositional mindfulness in our research than women. Zubair et al., (2018) found nonsignificant gender differences in mindfulness among Russian students; while male students reflected higher mindfulness as compared to women among Pakistan students [43]. 

We used self-assessment questions to measure subjective physical and mental health. Students rated their mental health status significantly worse than their physical health status in the current study. However, they felt the narrowing of their social relationships significantly worse than their physical health and mental health. University students are one of the most active groups of society. Lack of social contact has affected them sensitively. The most frequent changes in students’ behavior were more social distancing, loss of routine and social connections, more changes in education, and less going out in leisure time. Concerning physical health status, females reported significantly worse mental health and evaluated the narrowing of social relationships significantly worse than males. Conversano et al., (2020) found that gender and lockdown duration were good predictors of higher distress among Italians. The female gender seems to be solidly related with higher psychological distress than the male gender [17]. Female gender is associated with a higher risk of psychiatric symptoms and/or low psychological well-being [10,43,44,45,46].

No significant difference of well-being level was found regarding marital status. Concerning marital status, perceived stress levels of married/cohabitated respondents did not differ from the group of singles. Wang et al. [47] and Tian et al. [48] had similar findings to ours. Kowal et al. [40] found that married and cohabitating people were less stressed than singles. Odriozola-González et al. [49] provided evidence that single people compared to married had higher scores on stress, depression, and anxiety. Married or cohabitated students did not differ statistically from singles in the examined subjective mental/physical health and social relationships. Regarding trait mindfulness, there was no significant difference between the marital status groups in our sample. Marriage does not affect mindfulness as a personality trait in our sample. Unfortunately, there is no literature on whether there is a difference in the level of mindfulness between married/cohabitated and single persons. Erus (2020) found a correlation between mindfulness, interpersonal mindfulness in marriage, emotional intelligence, marital adjustment, and subjective well-being [50]. 

Sport plays a vital role not only in individual health but also in shaping wider society, it can improve general well-being. In our research, we found a significant difference between athletes and non-athletes regarding well-being score. Exercise sport regularly showed a significant correlation with well-being in other previous studies [33,34,35]. McMahon et al. [51] found that participation in sport/physical activity was strongly associated with a higher level of well-being and mental health. Jenkins et al. [52] (p. 2) noted “Emphasising PA as a buffer against psychological distress and preserving psychological well-being stems from a robust evidence base and has been advocated internationally as a way to reduce the negative impacts of the pandemic”. The results of Jenkins et al. [53] indicated that physical activity was positively associated with psychological well-being. 

The mean score of trait mindfulness was significantly higher for athletes too in our sample. Roberts and Danoff-Burg [53] found in their cross-sectional study that individuals who have higher mindfulness levels report better perceived health. They also found that daily physical activity level has a significant and positive association with mindfulness, as well as the extent to which physical activity was enjoyed and the number of days reported to be physically active in the past week. Regarding perceived stress total score, no significant difference was found between athletes and non-athletes in our sample. During the first wave of restrictions, sporting activities were limited to outdoor or home-based activities, so we assume that those who had exercise sport (mainly team sports) regularly before the curfew felt restricted, so this led to anxiety and stress.

Athletes had significantly better subjective physical health status than non-athletes, but athletes felt the narrowing of social relations more sensitively than non-athletes. However, no significant difference was found regarding mental health status. Previous studies have shown the positive effects of sports on achieving good physical, mental health, and psychological well-being [54,55,56,57]. Our study confirmed the positive impact of sports on quality of life by demonstrating this effect in a life situation that is especially stressful on mental and physical health. The protective effect of sports activity on mental health needs more detailed analysis and should be the subject of further publications. To explain the results accurately, it would be necessary to know what sport and which intensity the athlete group performed before curfew. Students’ mental health during the physical cessation of public life depended significantly on the level of changing their usual daily routine. With regards to the quick and radical changes in teaching and learning process and methods, studying at home produced worry about the learning activities (practical work, seminars, and lectures, absolving the exams, etc.). The majority of the students did not find the technical challenges disturbing, but students who answered to be stressed about technical challenges felt their subjective physical and mental health significantly worse and they detected the narrowing of social relationships more significantly, and felt significantly higher stress level and worse well-being level. Those who were not stressed about the technical requirements of learning had a significantly higher mean score of trait mindfulness. Students with positive or neutral attitudes towards absolving practical lectures online had higher values on the life quality and mental scales than students who reported difficulties. Fialho et al., (2021) measured with a cross-sectional online survey in Germany 5021 students at four universities and found that 54% of survey participants felt that the university workload had significantly increased since the COVID-19 pandemic. A positive association between perceived study conditions and depressive symptoms was found, and results of the linear generalized linear regression suggest that better student mental well-being was related to higher confidence in completing the academic semester [58]. Szlamka et al., (2021) reported regarding the specialist online counseling program from the Eötvös Lóránd University (ELTE) in Hungary that the clients had challenges with the distance learning, they experienced an increased workload, while not having clear channels of communication with other students and teachers [59]. Végh et al. measured 147 high school and 58 academic teachers with an online questionnaire during the first wave of COVID-19 in Hungary and asked them about their emergency remote-teaching habits and techniques. They found that academics were more likely to maintain interactions during their courses, and they preferred to make their own material for the lessons, while high-school teachers more often borrow material from pre-existing sources. The authors noted that over the past two decades, there has been a shift in university education, with blackboard education increasingly being replaced by digital (PowerPoint) presentations in Hungary [60]. In any case, we can state that the teaching style, personal preferences, and personality of the academics have a great influence on how the students coped with this special educational situation. 

## 5. Conclusions

Social isolation during the first wave of the COVID-19 pandemic negatively affected students and changed their lives significantly in Hungary. Students are an interesting group of people for researchers, as they have never experienced social disasters before. We used validated psychological scales to measure epidemic-related mental effects, including perceived stress levels, trait mindfulness, and well-being. We found a positive correlation between dispositional mindfulness and well-being, a negative correlation between dispositional mindfulness and perceived stress, and a negative correlation between well-being and perceived stress. We measured the correlation of the scales by gender and sporting attitude, and interesting differences between the established groups were found. Marital status seems not to be a protective factor in our higher education student sample. The pandemic affected students negatively and changed their life significantly.

### Limitations 

The included sample is non-representative, and the sample is collected from only one university (University of Pécs, Hungary). The findings are limited to a special group of the population, namely higher education students. The research focused on a special period during the first wave of the COVID-19 pandemic. These results cannot directly be extrapolated to other groups of the population or other nations. Our findings may be biased due to self-selection and subjective rating by respondents. Our sample had a higher proportion of female respondents. The study achieved a relatively large sample size and reached a wide range of students from different faculties at the university. The strengths of the study are the validated, internationally widely-used measure instruments scales and the sample size, which is relative high.

The research team assessed data from three COVID-19 waves of representative Hungarian population and university students, which not only provides a cross-sectional picture of the mental and physical health of the study groups in this specific situation but also provides an opportunity to identify trends. Students were negatively affected by the pandemic period, so it is important to implement activities and services at an institutional level to provide support to students that help address concerns related to the pandemic. Psychological interventions and trainings should be implemented to reduce the psychological harm caused by the COVID-19 epidemic.

## Figures and Tables

**Table 1:** Mean values; standard deviation; significance of MAAS, PSS-14, and WHO-5 total score; subjective physical and mental health; subjective rating of narrowing of the social relationships; stress about technical and communication changes in online courses regarding total, gender, marital status, and sporting habits.

		Gender			Marital Status			Sporting Attitude			Stress about Technical Challenges of Online Learning			Stress about the Communication with Professors and Fellow Students		
	Total	Male	Fe-male	Statistics/*p* Value	Married/Cohabited	Single	Statistics/*p* Value	Athlete	Non-Athlete	Statistics/*p* Value	Yes	No	Statistics/*p* Value	Yes	No	Statistics/*p* Value
**Subjective physical health**	3.49 ± 0.98	3.49 ± 0.99	3.49 ± 0.99	*p* = 0.929; Z = −0.089	3.47 ± 0.99	3.5± 0.98	*p* = 0.601; Z = −0.524	3.61 ± 0.96	3.20 ± 0.96	*p* < 0.001; Z = −5.623	3.33 ± 1.00	3.56 ± 0.96	*p* < 0.005; Z = −2.798	3.28 ± 1.01	3.62 ± 0.94	*p* < 0.001; Z = −4.702
**Subjective mental health**	3.01 ± 0.99	3.20 ± 0.99	2.96 ± 0.99	*p* = 0.003; Z = −2.924	3.07 ± 1.00	2.97 ± 0.99	*p* = 0.268; Z = −1.108	3.03 ± 1.01	2.98 ± 0.96	*p* = 0.482; Z = −0.703	2.65 ± 0.99	3.15 ± 0.95	*p* < 0.001; Z = −6.907	2.65 ± 0.96	3.25 ± 0.94	*p* < 0.001; Z = −8.472
**Subjective rating of narrow the social relations**	3.83 ± 1.63	3.59 ± 1.35	3.90 ± 1.22	*p* = 0.006; Z = −2.730	3.78 ± 1.23	3.87 ± 1.28	*p* = 0.193; Z = −1.303	3.92 ± 1.24	3.62 ± 1.29	*p* = 0.001, Z = −3.39	4.10 ± 1.13	3.72 ± 1.29	*p* < 0.001; Z = −3.981	4.05 ± 1.17	3.69 ± 1.29	*p* < 0.001; Z = −4.131
**PSS−14 total score**	31.4 ± 10.35	27.7 ± 10.19	32.5 ± 10.16	*p* < 0.001; Z = −5.703	30.78 ± 10.30	31.9 ± 10.37	*p* = 0.114; Z = −1.579	31.0 ± 10.35	32.43 ± 10.32	*p* = 0.101; Z = −1.641	35.6 ± 9.74	29.5 ± 10.06	*p* < 0.001; Z = −7.832	35.7 ± 9.81	28.5 ± 9.70	*p* < 0.001; Z = −9.667
**MAAS total score**	58.0 ± 12.50	60.9 ± 12.10	57.3 ± 12.51	*p* < 0.001; Z = −3.589	58.53 ± 12.64	57.7 ± 12.41	*p* = 0.378; Z = −0.881	58.7 ± 12.68	56.51 ± 11.96	*p* = 0.012, Z = −2.498	54.5 ± 12.64	59.6 ± 12.13	*p* < 0.001; Z = −5.254	54.5 ± 12.61	60.5 ± 11.85	*p* < 0.001; Z = −6.301
**WHO−5 score**	6.72 ± 3.24	6.92 ± 3.34	6.67 ± 3.34	*p* = 0.339; Z = −0.957	6.94 ± 3.42	6.56 ± 3.09	*p* = 0.217; Z = −1.235	7.03 ± 3.27	6.00 ± 3.04	*p* < 0.001; Z = −4.349	5.72 ± 3.19	7.17 ± 3.16	*p* < 0.001; Z = −6.125	5.77 ± 3.21	7.36 ± 3.10	*p* < 0.001; Z = −6.990
**N**	827	179	648		350	477		579	248		255	572		333	494	

**Table 2:** Mean values and standard deviation of MAAS, PSS-14, WHO-5 total score, subjective physical and mental health, and subjective rating of narrowing of the social relationships regarding absolving of theoretical and practical lessons during the distance learning.

	What Do You Think about the Learning Process Is Like for Practical Subjects (Seminars, Practical Lessons) in Form of Distance Learning						In Case of Theoretical Lectures Do You Consider that Learning the Course Material Was Successful during Distance Learning			
	It Was Harder and Much More Difficult to Acquire Practical Knowledge because It Was Shared Theoretically what You Should Do in Practice	To Learn the Practice only in Theory Is much More Difficult, because It Requires Independent Practice and Training at Home	Practicing at Home Is an Unfulfillable Task (Because of the Lack of Staff and Equipment).	I Find Easier to Absolve the Practice-Based Courses this Way	DO Not Feel Any Changes Compared to Previous Experience (Personal Attendance)	Can Not Decide the Question	To Learn the Theoretical Subjects Is more Difficult Because It Takes More Time to Process The Material	Learning Online Is „Easier, because Online Recorded Lectures can Be Replayed	Do not Feel Any Changes Compared to Previous Experiences	Can Not Decide the Question
**Subjective physical health (mean ± sd)**	3.47 ± 0.97	3.40 ± 1.06	3.39 ± 0.91	3.75 ± 0.86	3.56 ± 0.957	3.65 ± 1.05	3.37 ± 0.98	3.54 ± 0.99	3.56 ± 0.93	3.72 ± 1.01
**Subjective mental health (mean ± sd)**	2.91 ± 0.968	2.93 ± 1.036	2.81 ± 0.97	3.54 ± 0.92	3.28 ± 1.01	3.26 ± 0.88	2.65 ± 0.95	3.32 ± 0.95	3.31 ± 0.93	3.07 ± 0.88
**Subjective rating of narrow the social relations (mean ± sd)**	4.031 ± 1.204	3.89 ± 1.155	4.01 ± 1..22	3.60 ± 1.287	3.34 ± 1.37	3.40 ± 1.36	4.12 ± 1.14	3.57 ± 1.30	3.63 ± 1.38	3.78 ± 1.11
**PSS-14 total score (mean ± sd)**	33.52 ± 9.35	31.63 ± 9.770	34.88 ± 10.42	25.63 ± 10.59	26.08 ± 10.24	27.77 ± 10.01	35.22 ± 9.43	28.45 ± 10.18	27.85 ± 10.04	31.40 ± 9.86
**MAAS total score (mean ± sd)**	58.044 ± 12.71	57.23 ± 12.530	56.07 ± 11.42	59.07 ± 12.11	59.94 ± 11.86	61.04 ± 14.11	56.01 ± 12.81	59.56 ± 12.09	59.86 ± 12.33	59.40 ± 11.46
**WHO-5 score (mean ± sd)**	6.51 ± 3.11	6.61 ± 3.070	5.67 ± 3.12	8.96 ± 3.32	7.49 ± 3.39	7.25 ± 3.09	5.67 ± 2.93	7.69 ± 3.25	7.62 ± 3.24	6.59 ± 3.02
**N**	294	168	141	52	100	72	353	245	160	69

## Data Availability

The dataset supporting the conclusions of this article is available from the corresponding author on reasonable request.
